# There are no alternative hypotheses in tests of null hypotheses

**DOI:** 10.3389/fpsyg.2025.1708313

**Published:** 2025-11-04

**Authors:** Denis Cousineau

**Affiliations:** École de psychologie. Université d'Ottawa, Ottawa, ON, Canada

**Keywords:** null hypothesis significance testing (NHST), NHST controversy, alternative hypothesis, Statistical analysis, fallacies

## Abstract

Null hypothesis statistical testing (NHST) is typically taught by first posing a null hypothesis and an alternative hypothesis. This conception is sadly erroneous as there is no alternative hypothesis in the NHST. This misconception generated erroneous interpretations of the NHST procedures, and the fallacies that were deduced from this misconception attracted much attention in deterring the use of NHST. Herein, it is reminded that there is just one hypothesis in these procedures. Additionally, procedures accompanied by a power analysis and a threshold for type-II errors are actually a different inferential procedure that could be called dual hypotheses statistical testing (DHST). The source of confusions in teaching NHST may be found in Aristotle's axiom of excluded middle. In empirical sciences, in addition to the falsity or veracity of assertions, we must consider the inconclusiveness of observations, which is what is rejected by the NHST.

## Introduction

It is very common to see the description of a *t* test with the following hypotheses:


$$\begin{array}{ll} H_{0}: & \mu_{1}=\mu_{2}\\ H_{1}: & \mu_{1}\neq \mu_{2} \end{array}$$

These are the very first step shown and introduced when teaching this procedure, and already, half of it is wrong! It is then no surprise that so many misconceptions and so-called fallacies were derived from this incorrect conceptualization (starting with Rozeboom, 1960; also see Greenland et al., 2016; Trafimow, 2003, among many others).

This misconceptualization is general and is also present when teaching ANOVAs (“$H_{1}:\mu_{i}\neq \mu_{j}$ for at least one pair *i, j*”) and other null hypothesis statistical tests (NHST). It is vastly widespread, being found in many statistical teaching aids and textbooks to many disciplines (to name a few, Field, 2009; deGroot and Schervish, 2012; Gravetter and Wallnau, 2017; Agresti, 2021; for a review, see Cassidy et al., 2019), it has a long history (already Fisher, 1935, discusses it) and is enduring (Lytsy et al., 2022), affecting students and their educators equally as well as statisticans (Haller and Krauss, 2002; Lecoutre et al., 2003).

Fisher (1925), who created or formalized many of these procedures, never appealed to an alternative hypothesis. He made his view clear, stating that “Every experiment may be said to exist only in order to give the facts a chance of disproving the null hypothesis”. (Fisher, 1935, p. 16; also see Lehmann, 2011), adopting a stance similar to Popper's falsificationism (Popper, 1963). The alternative hypothesis was invented by Neyman and Pearson (1928) for a different purpose (see last section); Fisher disavowed its usage (Fisher, 1935; Denis, 2004; Cohen, 1992b).^1^

In what follows, we first summarize a typical null hypothesis test procedure, the well-known *t* test, highlighting what are the necessary ingredients to perform this test. As will be seen, nowhere is information from a so-called alternative hypothesis needed. We next conjecture that the source of the error is to be found in Aristotle's conception of logic as being two-valued only. We argue that adopting an approach that eliminates alternative hypotheses would enhance considerations given to the notion of evidence-based inferences. Third, we derive common errors that arose from this misconception. All these errors, occurring in undergraduate students and well-trained researchers, would simply not exist if the alternative hypothesis had never been taught. Finally, as promoted by Cohen (1969, 1992a), it is possible to test two hypotheses, but the resulting framework is quite different from the tests of null hypotheses; we end by clarifying the distinctions between the two procedures.

## The ingredients of a null hypothesis statistical test

To illustrate the absence of an alternative hypothesis in NHST, we comment on a single instance, the *t* test on two independent groups. The conclusion reached at herein is the same for any other test of a null hypothesis such as the ANOVAs. The *t* test can be derived in many ways; herein, we proceed via a model comparison approach. Let us define the following two models, the second being a restricted version of the first:


$$\begin{array}{ll} M_{a}: & X_{ij}\sim N\left(\mu_{i},\sigma \right)\\ M_{b}: & X_{ij}\sim N\left(\mu ,\sigma \right), \end{array}$$

in which *X*_*ij*_ denotes a realization of the dependent variable in group *i* (*i* = 1 or 2) for participant *j* (*j* = 1, …, *n*_*i*_), N denotes a normal distribution with parameters μ and σ. In the more general model *M*_*a*_, μ_*i*_ are allowed to differ between the two groups, but in the restricted model *M*_*b*_, both μ_1_ and μ_2_ are restricted to be equal (noting μ without a subscript). In both groups, the standard deviation is the same (from the so-called *homogeneity of variance assumption*).

As the various μs are parameters, they require estimators. The ones that maximize the likelihood of the models,


$$\begin{array}{ll} \ell_{a}\left(\mu_{i},\sigma |X_{ij}\right) & =\overset{2}{\underset{i=1}{\prod }}\overset{n_{i}}{\underset{j=1}{\prod }}f\left(X_{ij}|\mu_{i},\sigma \right)\\ \ell_{b}\left(\mu ,\sigma |X_{ij}\right) & =\overset{2}{\underset{i=1}{\prod }}\overset{n_{i}}{\underset{j=1}{\prod }}f\left(X_{ij}|\mu ,\sigma \right), \end{array}$$

where *f* is the PDF of the normal distribution, are the means in each group (μ_*i*_ estimated by ${\bar{X}}_{i}$ for *M*_*a*_) or the grand mean (μ estimated by $\bar{\bar{X}}$ for *M*_*b*_). The results are denoted as $\ell_{a}^{*}$ and $\ell_{b}^{*}$.

Minus twice the log of the maximized likelihood ratio (λ^*^) simplifies to the usual *t* statistic squared,


$$\begin{array}{l} -2\log\left(\lambda^{*}\right)=-2\log\left(\frac{\ell_{b}^{*}}{\ell_{a}^{*}}\right)\\ \;={\left(\frac{{\bar{X}}_{1}-{\bar{X}}_{2}}{\sqrt{2}s_{p}/\sqrt{ñ}}\right)}^{2} \end{array}$$

where *s*_*p*_ is the pooled standard deviation and ñ is the harmonic mean of the two groups' sample sizes.

The likelihood ratio test (Wilks, 1938) states that minus twice the log ratio of two likelihoods for maximally likely models, one being a restricted version of the other, follows a chi-square distribution with degrees of freedom given by the difference in the number of maximized parameters in both models (here 2 − 1 = 1). Wilks's result is asymptotic (infinitely large *n*); Fisher (1925) generalized this result for small *n* with the *F* distribution. As known, the square root of variates following the *F* distribution with 1 degree of freedom at the numerator is a *t* distribution. Previously, Gossett (Student, 1908) found the *t* distribution using an independent approach.

In summary, this test compares the model where the means are as observed in both groups relative to the model where the mean is the same in both groups. The second model will necessarily have a poorer fit, but the critical question is to assess how detrimental the restriction is. Using a decision threshold α, it is possible to determine limits beyond which the restriction is too severe to be plausibly maintained.

The final result of this procedure is a rejection region surrounding the null and delimited by boundaries *t*_left_ = *t*_*n*_1_+*n*_2_−2, α/2_ and *t*_right_ = *t*_*n*_1_+*n*_2_−2, 1−α/2_ whose extent is based on the decision threshold α and the sample size *n*. Nowhere are the specifications provided by the alternative hypothesis used in this whole procedure. Therefore, why postulate one?

## The source of the confusions

The reason why so many students, researchers and teachers alike feel an urge to add an alternative hypothesis may be related to how logical statements are conceived. For many, a statement is either true or false, but no other states are conceivable. This conception dates back to antiquity. For example, Aristotle assumed that if two propositions are in opposition (i.e. where one proposition is the negation of the other, that is, mutually exclusive), then one must be true, and the other must be false (Aristotle, ca. 340 BC; English translation 2015). This came to be known as the *axiom of the excluded middle*.

This position may be sensible in mathematics; however, it poorly fits how the acquisition of knowledge—and science generally speaking—progresses. In the empirical sciences, it is not possible to prove that a theory is true or false. Thus, the intrusion of the axiom is dubious. In an empirical investigation, a sample is gathered and evaluated with respect to a *status quo ante* position, that is, a position that lacks a novel effect. This evaluation could provide little support for this *no-effect* position where the strength of this misfit is assessed, for example, with a *p* value. However, a nonextreme *p* value says nothing with regard to the *status quo*. Asked “Should we abandon the *status quo*?”, the correct answer in this case would be “We still do not know” because the sample does not provide strong-enough evidence.

In this view, the acquisition of knowledge is build from a preliminary *state of unknowing*. When rejecting the null, we decide to leave this state for a state excluding the null. On the other hand, not rejecting the null means that we remain in the state of unknowing. In Howell's words, we must *suspend any judgment* (Howell, 2010, p. 94).

This principle summarizes *evidence-based research*. Either the evidence is sufficient to exclude the null or it is inconclusive. Here, an inconclusive state does not mean that we move to a state including the null state; rather, the state of knowledge is stalled and unchanged, because of a lack of evidence.

Alternatively, confusion may have to do with the *modus tollens*. For the premisse “If the data are congruent with $H_{0}$, then *p*>0.05”, we can conclude that “The data are not congruent with $H_{0}$” when “*p* is not larger than 0.05”. However, when $H_{1}$ is defined as the negation of $H_{0}$, then the conclusion rapidly becomes “The data are congruent with $H_{1}$”. Sadly, many things are wrong here, including the premisse. It should read “If the *population* is congruent with $H_{0}$, then *p*>0.05 *most of the time*”.

## Consequences of postulating an alternative hypothesis

Assuming the existence of an alternative hypothesis has consequences, and these consequences are all negative. Four are hightlighted here.

### Accepting the null

Statistics instructors spend numerous hours dispelling this incorrect conclusion. Why is it so recurrent and so difficult to atone? *The problem of how to interpret a nonrejected null hypothesis has plagued students in statistics courses for over 75 years* (Howell, 2010, p. 93). Despite numerous discussions and warnings (among others, Lecoutre et al., 2003), a recent survey suggests that accepting the null is still widely performed by researchers (Edelsbrunner and Thurn, 2024).

The persistence of this error may be related to a framing effect: By introducing two propositions that are in opposition, we place the student in a *logic* mode of thinking. In this mode, if A is not true, then its opposing statement *has* to be true. In this mode, what is a nonacceptation of the alternative if not an acceptance of the null?

Not teaching $H_{1}$ would avoid this dichotomized mode of thinking and more easily let the concept that if $H_{0}$ cannot be rejected, it is because the *data* are inconclusive (see Dushoff et al., 2019, for a similar argument).

### Misinterpreting the *p* value

Many come to the false conclusion that the *p* value is the probability of the null hypothesis (Cohen, 1992b). When the only visible outcome of the procedure is with regard to rejecting the null or not, the probability of the null is the only thing that comes to mind. If the NHST is presented without an alternative hypothesis, with a focus on obtaining evidence for rejecting the null hypothesis, then the *probability of the evidence* should come to mind, which is much closer to the true definition of the *p* value. The probability of the evidence places the focus on the data observed. Consequently, realizing that it is conditional on the null model assumed is a simple extension: the *p* value is the probability of the evidence assuming the null model.

### Appealing to a possible type-II error

It is frequent to read research articles in which the authors report a nonsignificant result and then appeal to a possible type-II error (deciding not to reject the null when it is false). The correct conclusion being that the data are inconclusive, how can a lack of conclusion be an erroneous conclusion?

Appealing to a possible type-II error shows that the outcome of the procedure is poorly understood. Many other possible interpretations are possible. For example, (*i*) the sample size may be too small to detect anything (Cohen, 1992a); (*ii*) the true effect might be non-null but so small that the experiment lacks the necessary sensitivity (Lykken, 1968); and (*iii*) the controls exercized on the sampled groups may be insufficients to bring forward the difference (Wilson Van Voorhis and Morgan, 2007).

A properly calibrated statistical procedure comes with a certain guarantee: the probability of error when a decision is made has a knwown magnitude. In NHST, this probability is adjusted with the decision threshold α. In NHST, there is no way to know the probability of a type-II error as there is no decision threshold for errors of this type (this second probability is usually represented with β in other inferential frameworks, e.g., the non-equivalence tests and the dual-hypotheses tests; see next section).

A type-II error may occur when a decision is endorsed. Not endorsing a position cannot result in an error. *Suspending our jugdment* is not a judgment. NHST is not designed to provide support to the null hypothesis. Hence, if the purpose is to support the null, do not use a NHST procedure.

### Overinterpreting confidence intervals

The confidence intervals are often conceived as alternative but equivalent representations of the NHST. This is not exactly the case. A confidence interval of a difference for example provides a zone in which all the population differences of size δ would not be rejected, if tested in a null hypothesis of the sort $H_{0}:\mu_{1}-\mu_{2}=\delta$. This zone can be called a *compatibility zone* (Amrhein et al., 2019; Wasserstein and Lazar, 2016). Population differences outside this zone are said to be incompatible with the observed data whose difference is $d={\bar{X}}_{1}-{\bar{X}}_{2}$.

**Figure 1:**
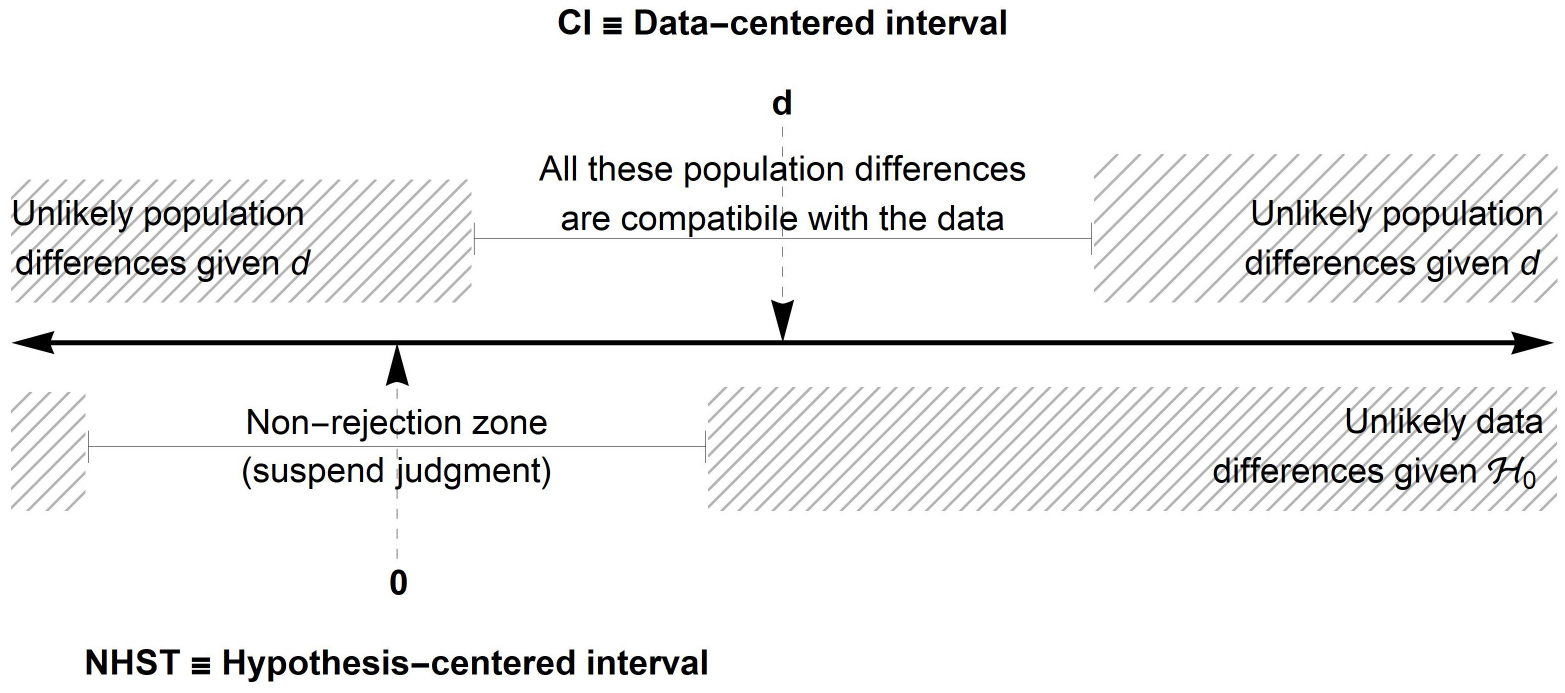
Illustration of an interval based on the null hypothesis (lower half) and an interval based on the observed difference *d* (upper part). Note: 0 is the population difference hypothesized in $H_{0}$; *d* is the observed difference. In this illustration, the observed difference *d* is located in the rejection zone so the null hypothesis $H_{0}$ can be rejected with a given decision level α.

One way to illustrate the two approaches is to realize that the confidence intervals provides an interval centered on the observed statistic whereas NHST offers an interval centered on the null hypothesis. Figure 1 illustrates these two intervals.

When 0 (or the value hypothesized by $H_{0}$) is included in the confidence interval, it does not mean that we accept $H_{0}$. It means that $H_{0}$ is *one possible interpretation* compatible with the data.

With confidence intervals, assigning a probability to a specific population parameter value is not possible. Therefore, accepting a specific population value is unwarranted because it is not possible to know the risk of an error to such a conclusion.

Sadly, many erroneous interpretations of confidence intervals abound among trained researchers and authors (Hoekstra et al., 2014). To be correct, the confidence interval must be conceptualized as a compatibility interval whereby differences outside the interval are incompatible with the observed data (Amrhein et al., 2019).

### Is the alternative hypothesis a questionable research practice?

Questionable research practices were first defined by Ioannidis (2005). Collectively, these practices deteriorate the quality and credibility of research even if most of the time, the authors are unaware of their presence (Sijtsma, 2016). Flake and Fried (2020) argued for the importance of defining the construct used in questionaires. For example, a researcher should be able to answer questions such as *What is your construct?, Why did you select this measurement?* Although they designed the questions to questionnaires and their items, the same questions can be made with respect to the framework used for statistical inference: *Why did you choose the NHST?* and *Why did you select the* *p-value for your inference*? We could also add: *How will you interpret that measure?* In the absence of a clear line of interpretation supported by the framework, unfounded conclusions can be drawn, reducing the credibility of the tool used.

## Power planning with NHST

Cohen (1969, 1992a,b), for many decades, observed that typical sample sizes in the social sciences where too often very small and consequently had little chance of rejecting the null hypothesis when it is false. This notion is call the statistical power of a design. To improve statistical power, he suggested that, during the planification stage, experimenters settle on one specific alternative hypothesis (e.g., $H_{1}:\mu_{1}=\mu_{2}+\Delta$ for a one-directional test, or $H_{1}:\mu_{1}=\mu_{2}\pm \Delta$ for a two-directional test; Δ≠0). Using this specific alternative, it is then possible to find a sample size such that –simultaneously– the probability of being outside the rejection zone of $H_{0}$ when it is true is a desired α level and the probability of being inside the rejection zone of $H_{0}$ when $H_{1}$ is true is a desired 1−β level (Cohen suggested using α = 0.05 and 1−β = 0.80). Once the sample size is set, Cohen would simply forget $H_{1}$ and continue with a regular NHST.

This approach is now commonly used in planning a design and has been very efficient in improving statistical power in the psychological and social sciences. It was inspired by the Dual hypotheses testing used in Neyman and Pearson, as seen below, and became a practical approach with the advent of noncentral distributions that were being discovered between the 1930s and the 1950s (e.g., Johnson and Welch, 1940).

## Dual hypotheses statistical testing (DHST)

Neyman and Pearson (1928, 1933) considered an approach with two hypotheses. In this view, the alternative hypothesis is likewise a pointwise hypothesis, such that


$$\begin{array}{ll} H_{0}: & \mu_{1}-\mu_{2}=0\\ H_{1}: & \mu_{1}-\mu_{2}=\Delta \end{array}$$

The analyst must set a decision threshold α but also a decision threshold β. With this dual testing procedure, it is possible to reject $H_{0}$, which says that evidence favors $H_{1}$ over $H_{0}$ and vice versa, it is possible to reject $H_{1}$, which says that evidence favors $H_{0}$ over $H_{1}$. Thus, $H_{0}$ can be accepted (Cohen, 1992b, p. 1308).

As a consequence, there is a possibility that a type-II error occurs when $H_{0}$ is rejected. It is also possible that a type-I error occurs when rejecting $H_{1}$. Both error probabilities are adjusted to acceptable levels by setting α and β as desired. As suggested by the features of these inferential frameworks listed in Table 1, the DHST can be seen as a combination of NHST and non-equivalence hypothesis testing (Lakens et al., 2018).

**Table 1:** Comparison of Null-hypothesis statistical tests (NHST), Dual-hypotheses statistical tests (DHST), and non-Equivalence hypothesis statistical tests (¬EHST) frameworks in the context of two-group comparisons.

**Feature**	**NHST**	**DHST ^†^**	**¬EHST ^‡^**
Hypothesis	$H_{0}:\mu_{1}-\mu_{2}=0$	$H_{0}:\mu_{1}-\mu_{2}=0$ $H_{1}:\mu_{1}-\mu_{2}=\Delta$	$H_{1}:\mu_{1}-\mu_{2}=\pm \Delta$
Error Control	type I: α	type I: α type II: β	type II: β
Possible decisions	*reject $H_{0}$* *status quo*	*reject $H_{0}$* *reject $H_{1}$*	*status quo* *reject $H_{1}$*

^†^ DHST are necessarily directional, “facing” each other. For example, if δ is larger than 0, then $H_{0}$ is tested in the positive direction and $H_{1}$ is tested in the negative direction. ^‡^ ¬EHST are necessarily tested toward the value in-between −Δ and +Δ. In this table, the shortcut “ = ±Δ” means greater or equal to +Δ or smaller or equal to −Δ.

## Conclusion

Discussing with colleagues that there is no alternative hypothesis in NHST, many simply replied that as long as it helps the student understand the logic of statistical testing, teaching $H_{1}$ is inconsequential. Instead, we believe that many errors and misconceptions arise from the erroneous teaching of $H_{1}$ and that the students would be better without this concept. It it more appropriate to say that after a non-significant result, “we still don't know whether $H_{0}$ should be abandoned or not”, or “we must suspend our judgment until more decisive data are collected” (Jones and Tukey, 2000, p. 412).

As argued in this text, the many errors that are triggered by the erroneous presence of an alternative hypothesis in the NHST are actually *language* errors built on cognitive limits and approximate guesses from the learners. These errors could be reversed by providing a deeper understanding of the NHST's inner gears and meanings (e. g., Wasserstein and Lazar, 2016) or teaching Bayesian statistics in parallel (Lecoutre, 2006). However, it seems easier to just *obliterate* the source of the error: there is no alternative hypothesis.

We urge instructors of statistics to stop including an alternative hypothesis when presenting NHST. It is possible, see Howell, 2010 (or in french, Cousineau, 2009). This error is creating considerable harm and confusion, and taken literally, results in fallacies. Removing a single line (“$H_{1}:\mu_{1}\neq \mu_{2}$”) which is not used anywhere, will minimize or eliminate the many misconceptions that are triggered by it.

## Footnotes

^1^“It might be argued that if an experiment can disprove the [null] hypothesis [...], it must therefore be able to prove the opposite hypothesis [...]. But this last hypothesis, however reasonable or true it may be, is ineligible as a null hypothesis [...] because it is inexact” (Fisher, 1935, p. 16). By *inexact*, Fisher meant that it is not a pointwise hypothesis. Note that Fisher commonly disavowed other researchers' proposals.
